# Deciphering the Roles of Thiazolidinediones and PPAR**γ** in Bladder Cancer

**DOI:** 10.1155/2017/4810672

**Published:** 2017-02-28

**Authors:** Melody Chiu, Lucien McBeth, Puneet Sindhwani, Terry D. Hinds

**Affiliations:** ^1^Center for Hypertension and Personalized Medicine, Department of Physiology & Pharmacology, University of Toledo College of Medicine, Toledo, OH 43614, USA; ^2^Department of Urology, University of Toledo College of Medicine, Toledo, OH 43614, USA

## Abstract

The use of thiazolidinedione (TZD) therapy in type II diabetic patients has proven useful in the lowering of blood glucose levels. However, recent investigations have shown that there may be potential health concerns associated, including the risk of developing bladder cancer as well as complications in the cardiovasculature. TZDs are ligands for the nuclear receptor PPAR*γ*, and activation causes lipid uptake and insulin sensitization, both of which are critical processes for diabetic patients whose bodies are unable to utilize insulin effectively. Several studies have shown that PPAR*γ*/TZDs decrease IGF-1 levels and, thus, reduce cancer growth in carcinomas such as the pancreas, colon, liver, and prostate. However, other studies have shed light on the potential of the receptor as a biomarker for uroepithelial carcinomas, particularly due to its stimulatory effect on migration of bladder cancer cells. Furthermore, PPAR*γ* may provide the tumor-promoting microenvironment by de novo synthesis of nutrients that are needed for bladder cancer development. In this review, we closely examine the TZD class of drugs and their effects on PPAR*γ* in patient studies along with additional molecular factors that are positive modulators, such as protein phosphatase 5 (PP5), which may have considerable implications for bladder cancer therapy.

## 1. Introduction

The predominant type of bladder cancer diagnosed among individuals in the United States is urothelial (transitional cell) carcinoma [1]. Bladder cancer is the fourth most common type of cancer found among men in the United States and an important cause of death worldwide [2, 3]. In 2015 alone, the American Cancer Society predicted a total of 74,000 newly diagnosed cases and 16,000 deaths from bladder cancer in the United States [4]. The cause of bladder cancer appears to be multifactorial; both exogenous environmental and endogenous molecular factors may potentially play a role in cancer development [5, 6]. Environmental factors such as cigarette smoking and occupational exposure to chemical carcinogens are among the top risk factors; however, family history and genetics also increase the susceptibility to bladder carcinogenesis [7]. Moreover, evidence has suggested an association between diabetes mellitus and the increased risk of bladder cancer [8]. Rates of type II diabetes mellitus among adults and children have been continuously rising. In the 2014 National Diabetes Statistics Report, the Centers for Disease Control and Prevention estimated that 29.1 million people (9.3% of the population) are diagnosed with diabetes in the United States [9]. Worldwide, an estimated 382 million adults were diagnosed with diabetes in 2013 [10], with type II diabetes accounting for nearly 90–95% of these diabetic individuals [11].

There has been increasing evidence showing that antidiabetic TZDs are linked to the risk of bladder cancer as well as other complications such as cardiovasculature (CVD) events. TZDs, such as pioglitazone and rosiglitazone, are synthetic ligands of peroxisome proliferator-activated receptors gamma (PPAR*γ*) used in therapeutic treatments for patients diagnosed with type II diabetes mellitus [12, 13]. These ligands bind to PPAR*γ* and play a role in metabolism through induction of genes that control glucose and lipid uptake [13]. Through a series of metabolic pathways, PPAR*γ* also activates adipogenesis, which is the process of transforming a preadipocyte stem cell into fully mature adipocyte [14]. Eventually, this process reduces insulin resistance by assisting in glucose uptake [15]. Potentially, PPAR*γ* signaling in bladder cancer cells may provide a tumor microenvironment that allows for de novo lipogenesis for the use of increasing tumor mass and energy usage. However, the role of PPAR*γ* in bladder cells is unknown.

PPAR*γ* is expressed in white and brown adipose tissues as well as in the urinary bladder [16, 17]. More notably, high levels of PPAR*γ* are selectively expressed in the transitional epithelium of the ureter and urinary bladder, the area where bladder cancer typically arises. PPAR*α* is another member of the PPAR family that is expressed in the ureter and bladder epithelium, but at a significantly lower level compared to PPAR*γ* [17]. Despite the prominent differences between the two receptors, there has also been evidence depicting a degree of crosstalk between the receptors in urinary bladder epithelium. A combination of synthetic ligands, known as “dual-acting agonists,” includes PPAR*α* and PPAR*γ* agonists and has been shown to have a carcinogenic impact in rodents, primarily affecting the bladder epithelium [18]. In this review, we discuss the functions of PPAR*γ* and the effects of TZD therapy in the urinary bladder and to a lesser extent the role of PPAR*α*.

## 2. PPAR***γ*** Function

The PPAR*γ* gene is located on chromosome 3 in humans and is alternatively spliced to produce two major proteins; however, alternative usage of the promoter provides four different transcripts [19, 20]. The mRNAs of transcripts PPAR*γ*1, PPAR*γ*3, and PPAR*γ*4 result in identical protein products that we refer to as PPAR*γ*1. The protein product from the mRNA of PPAR*γ*2 is comparable to that of PPAR*γ*1; however, the product contains 30 additional amino acids located at the NH_2_-terminal region (reviewed in [20]) [21]. Not surprisingly, the isoforms have varying expression levels in cells; PPAR*γ*1 is expressed in nearly all cells, whereas PPAR*γ*2 is principally expressed in adipocytes [22]. However, it is unknown whether there is a difference in PPAR*γ*1 and PPAR*γ*2 expression levels in bladder cancer cells. PPAR*γ* is also involved in regulating inflammatory processes [23]. There is evidence that shows PPAR*γ* activation in endothelial cells reduces systemic inflammation [24]. While the role in adipocytes and insulin sensitivity is well understood, the effects of PPAR*γ* activation in many other cell types remain unclear including bladder cancer.

**Figure 1:**
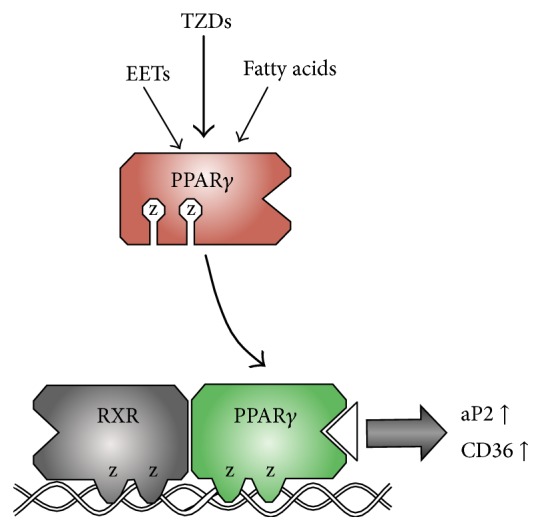
PPAR*γ* heterodimerizes with RXR for transcriptional regulation. PPAR*γ* ligands such as eicosanoids (EETs), fatty acids, or thiazolidinediones (TZDs) bind to PPAR*γ* to cause transactivation resulting in the binding to regulatory regions on DNA. PPAR*γ* combines with retinoid X receptors (RXRs) to form heterodimers, which together serve as transcriptional activators for various genes by binding to specific PPAR response elements (PPREs) in their promoters.

PPARs are ligand-activated transcription factors that belong to the nuclear receptor superfamily [22]. When a ligand binds to an isoform of the PPAR family, the receptor is activated, translocates to bind regulatory regions on DNA, and then combines with retinoid X receptors (RXRs) to form heterodimers (Figure 1). Consequently, these heterodimers serve as transcriptional activators for various genes by binding to specific PPAR response elements (PPREs) [13]. Of the PPARs, PPAR*γ* is found to have the highest expression levels in adipose tissue. Once activated in adipocytes by TZDs or natural ligands, such as essential fatty acids and eicosanoids [25], PPAR*γ* is involved in the secretion of adiponectin and leptin. These adipokines regulate insulin activity in peripheral tissues to maintain glucose sensitivity in the body. In addition, PPAR*γ* regulates genes involved in fatty acid transport, release, and storage by increasing expression of genes involved in fatty acid import such as cluster of differentiation 36 (CD36) and adipocyte protein 2 (aP2) [21, 26]; therefore, PPAR*γ* has a major role in lipid and carbohydrate metabolism.

TZDs have long been a common therapeutic method to treat patients with type II diabetes mellitus. TZDs are used to treat hyperglycemia and insulin resistance, lowering fasting blood glucose and insulin, as well as HbA1C levels [27]. Previously, up to 20% of antidiabetic medications prescribed in the USA were TZDs [28]. In the past, it has been shown that TZDs are effective in therapy as a second-line treatment after metformin, the current first-line agent in type II diabetes [27, 29]. They are high-affinity synthetic agonists of PPAR*γ* [12], and PPAR*γ* activation affects lipid metabolism and ultimately enhances lipid storage and promotes insulin sensitivity in adipose tissue, liver, and muscle [16, 23]. Despite many benefits, TZDs have also been shown to induce weight gain among diabetic patients on long-term therapy [30], which occurs from activation of adipogenesis and the expansion of fat cells. Of the TZD class, rosiglitazone and pioglitazone are the most prevalently used drugs in clinical settings [31]. Studies have reported the adverse health effects of these medications, including the possible risk of developing bladder cancer or cardiovascular events [12, 32, 33]. However, there is a conundrum for the effects of PPAR*γ* and its ligands in cancer. Several cancers have shown reduced growth with PPAR*γ* activation with the TZD troglitazone such as in carcinomas of the breast, kidney, liver, colon, pancreas, and prostate [34–39] as well as in non-small-cell lung cancer [40] and ACTH-secreting pituitary adenomas [41]. However, most of the antigrowth properties of TZDs have been with troglitazone and not pioglitazone or rosiglitazone. Rosiglitazone may be associated with a lower risk of breast cancer [42], thyroid cancer [43], and nonmelanoma skin cancer [44]. On the other hand, pioglitazone seems to be neutral or slightly (possibly not significant) associated with various cancers including bladder cancer [45], ovarian cancer [46], oral cancer [47], kidney cancer [48], and thyroid cancer [49]. Analysis of specific TZDs and their actions on growth and migration are important for understanding the impact they may have in a specific cancer.

Some TZDs have been shown to reduce levels of the insulin-like growth factor-1 (IGF-1) in the blood, which is a known growth factor that may induce cancer [50]. Plasma levels of IGF-1 and IGF binding protein-3 (IGFBP-3) have been shown to be an association with bladder cancer risk [51]. It is not known how PPAR*γ* affects the expression of IGF-1, IGFBP-3, or the IGF receptor (IGFR) in the bladder or differences among the TZD drug class. The use of pioglitazone, and not rosiglitazone, has been associated with an increased risk of bladder cancer in a population-based cohort study, suggesting the risk is TZD specific and not a particular class [52]. Investigations on the consequences of troglitazone, rosiglitazone, and pioglitazone on the IGF system in uroepithelial carcinomas may reveal differences between the drugs.

## 3. TZDs and Bladder Cancer

An interim longitudinal cohort study using the Kaiser Permanente Northern California Registry analyzed a sample size of 193,099 diabetic patients and observed a correlation between pioglitazone therapy and bladder cancer [12]. The increased dosage and duration of pioglitazone treatment show rises in bladder cancer incidence rates, with a 30% risk of developing bladder cancer among patients on pioglitazone therapy after 12–24 months. Furthermore, the risk increases to 50% for patients on pioglitazone therapy for 2 or more years [12]. In the 10-year follow-up, however, the statistical significance was not found while there was a numerical increased adjusted risk of 78% (0.93–3.4, 95% CI) for patients on pioglitazone treatment for 1.5–4 years [53]. Additionally, Hsiao et al. showed current users of both pioglitazone and rosiglitazone had increased risks of developing bladder cancer [32]. The correlation between pioglitazone and bladder cancer is consistent with the previous Kaiser cohort study. However, the use of rosiglitazone was not associated with an increased risk of bladder cancer in any analysis [52], but it has been linked to increased risk of cardiovascular events [54]. However, rosiglitazone was not increased in bladder cancer risk [55].

Pioglitazone may be the only TZD to enhance cases of bladder cancer, as results from the National Health Insurance Research Database (NHIRD) group also presented an association with uroepithelial carcinomas [32]. Through the NHIRD study, it was shown that increased exposure period to both pioglitazone and rosiglitazone is related to an increased risk of bladder cancer. Regardless of whether patients have been on pioglitazone or rosiglitazone treatment, the highest risk of bladder cancer is among diabetic patients with the longest exposure to either treatment. The NHIRD cohort showed the odds ratios for the risk of bladder cancer among diabetic patients on pioglitazone therapy in the exposure groups were 1.45 (<1 year), 1.74 (between 1 and 2 years), and 2.93 (2 or more years) [27]. Similarly, odds ratios for patients on rosiglitazone therapy were 0.98 (<1 year), 1.78 (between 1-2 years), and 2.00 (2 or more years) [31]. The increased duration of pioglitazone or rosiglitazone therapy is associated with increased risk of bladder cancer, with the highest risk among diabetic patients on therapy for 2 or more years [32]. However, this observation may only apply to specific TZDs and not all of them [29], as there appears to be a weaker association between bladder cancer and rosiglitazone.

There is some debate as to the association of TZDs with bladder cancer. Two meta-analyses show only moderate to no risk of developing bladder cancer. Monami et al. found that the overall risk of malignancies (regardless of location) was decreased by TZD treatments [56]. However, there was a numerical, but not statistically significant, increase in the risk of bladder cancer development from pioglitazone treatment (2.05 Mantel-Haenszel odds ratio, *p* = 0.12) but no association with rosiglitazone treatment (0.91, *p* = 0.62). Interestingly, the odds ratio was associated with a large confidence interval, 0.84–5.02, which the authors attributed to a small sample size, three studies, due to potential bias from incomplete disclosure of negative results. In addition, the second meta-analysis conducted by Bosetti et al. showed only a modest increased risk of developing bladder cancer when treated with TZDs for less than two years (relative risk 1.20, CI 1.07–1.34) [57]. There was a moderate increased risk for treatment longer than two years (relative risk 1.42, CI 1.17–1.72), which the authors led to claim that the short-term (less than two years) treatment with TZDs in type II diabetes mellitus might be worth the modest risk of developing bladder cancer.

## 4. PPAR***γ*** and Bladder Cancer

To provide a closer look at the impact of PPAR*γ* on bladder cell progression, Yang et al. analyzed samples of both benign bladder and bladder cancer mucosal samples by fluorescence in situ hybridization (FISH) assay for expression of PPAR*γ*, and the authors found 31% (8/21 samples) of the bladder cancer mucosal samples and 4.3% (1/23 samples) of benign bladder samples showed amplification [58]. In addition, lower levels of PPAR*γ* amplification were detected in non-muscle-invasive bladder cancer samples compared to muscle-invasive samples (16.7% versus 46.7%, resp.) [58]. Yang et al. also observed different rates of cell migration and invasion in various bladder cancer cell lines that have PPAR*γ* expression. The 5637 bladder cell line had a considerably higher mRNA and protein expression of PPAR*γ* compared to other bladder cancer cell lines such as UMUC-3. Moreover, the 5637 cancer cell line displayed higher cell migration and invasion than the UMUC-3 cell line [58]. Another study showed that the T24 bladder cancer cell line expresses PPAR*γ* and high levels of the nuclear receptor glucocorticoid receptor *β* (GR*β*), which also showed higher migration rates than the UMUC-3 cells that have low PPAR*γ* and GR*β* expression [59]. These results suggest that PPAR*γ* may be a potential biomarker of bladder cancer aggressiveness, where high levels of receptor expression correlate with higher rates of cancer cell migration and invasion.

Rosiglitazone treatments have been shown to have varying effects on 5637 and UMUC-3 cancer cells [58]. The 5637 bladder cancer cells display significantly enhanced cell migration and invasion with rosiglitazone treatment. On the other hand, there are minimal rates of cell migration and invasion in UMUC-3 cells, and rosiglitazone has less of an effect. The difference in the levels of PPAR*γ* expression between the two cancer cell lines may account for this observation, as the 5637 cell line has a considerably higher PPAR*γ* expression than UMUC-3 cell line [58]. Lubet et al. performed a series of experiments using rosiglitazone and hydroxybutyl(butyl)nitrosamine (OH-BBN), which is a carcinogen that is known to induce urinary bladder cancer in rats [13]. Interestingly, rats treated with rosiglitazone had 100% incidence of bladder cancer, while the untreated control group had a 57% incidence of bladder cancer. There were also increased levels of PPAR*γ* expression in the presence of rosiglitazone treatment compared to those that were not treated. Furthermore, rats that were exposed to OH-BBN and treated with the highest dosage of rosiglitazone have the highest incidence of bladder cancer. Rats on rosiglitazone therapy had earlier cancer onsets and larger tumor sizes in the bladders, and a dose-dependent response existed between rosiglitazone and bladder cancer incidence. TZDs may not have an effect in the earlier stages but may promote cancer progression at the later stages of bladder cancer [13]. However, it is important to note that in humans rosiglitazone has not been associated with higher risk, but this has been observed with pioglitazone. Regardless, decreasing PPAR*γ* expression may potentially alter bladder cancer migration and invasive abilities. Therefore, regulating levels of PPAR*γ* expression in the urinary bladder may have implications for targeting bladder cancer, particularly regarding metastasis and cancer cell progression.

## 5. An Independent Microenvironment through PPAR***γ***

In general, tumor development in the urinary bladder is dependent upon complex interactions with host molecular factors that are part of its surrounding microenvironment [60, 61]. Furthermore, there are signaling interactions of a certain level in the microenvironment that are capable of inducing malignant transformation of cells, such as factors that promote angiogenesis, abnormal development, and proliferation. Neoangiogenesis, or the formation of new blood vessels from preexisting vessels, is required for tumor growth, and vascular endothelial growth factor (VEGF) has been shown to play a critical role as a proangiogenic factor in bladder cancer progression [62]. VEGF-A is the primary proangiogenic factor that serves to maintain adequate levels of oxygen and nutrient supply in growing adipose tissue and is positively regulated by PPAR*γ* [63]. The levels of VEGF found in the urine and bladder tissue are significantly elevated in patients diagnosed with urinary bladder carcinoma compared to cancer-free patients [64]. Additionally, it has been shown that VEGF-A is found in bladder tumors and is upregulated in patients with invasive bladder cancer [65]. Potentially, VEGF-A may also be enhanced by PPAR*γ* in bladder tumor tissue consequently enhancing tumor growth and migration through angiogenesis. However, the specific TZDs that may enhance VEGF-A or if PPAR*γ* induces VEGF-A in bladder are yet to be determined.

In order to continue to proliferate indefinitely, cancer cells require molecular factors that increase both glucose uptake and rates of glycolysis for energy. Elevated rates of glycolysis produce higher amounts of lactic acid, and this pathway enhances lipogenesis through fatty acid synthase (FAS). FAS is the key enzyme involved in de novo synthesis of fatty acids for lipid storage, and high expression levels are frequently limited in tissues with lipogenic activity, such as adipose tissue and liver [66]. However, it has been shown that FAS is overexpressed in numerous human cancers, including bladder cancer, and its expression level is positively correlated with tumor progression [67]. Similar to FAS, fatty acid binding proteins (FABPs) are involved in lipid metabolism and facilitate the transfer of lipids, including lipid droplets for storage, across various cellular membranes and compartments [68–70]. Adipocyte-type FABP (A-FABP), also known as adipocyte protein 2 (aP2) and fatty acid binding protein 4 (FABP4), binds to long chain fatty acids and PPAR*γ* agonists [69]. These ligands bind and activate A-FABPs in the cytosol, and A-FABPs then transfer the ligands to PPAR*γ* upon entering the nucleus to drive adipogenic activities [71]. Unlike FAS, low expression levels of A-FABP are correlated with the progression of human bladder transitional cell carcinoma. When comparing specific types of bladder tumor tissue, A-FABP was mainly detected in cells that were papillary in origin and not invasive urothelial carcinoma [72]. Evidence suggests low-grade bladder tumors have higher levels of A-FABP compared to high-grade bladder tumors [73]. On the other hand, high expression of A-FABP has been observed in tongue squamous cell carcinoma [70]. The differences in tissue types, such as bladder and tongue, may partially account for the discrepancy in the effects of A-FABP expression.

Metabolic changes may occur in nonadipose tissues when they receive fatty acids released by hypertrophic dysfunctional adipose tissue, commonly seen among obese and type II diabetic patients [74]. Nonadipose tissues are not equipped with adequate cellular machinery for excessive amounts of lipid deposits. Therefore, an overload of lipids in these tissues causes a series of organ-specific toxic reactions and results in lipotoxicity, which is lipid-induced metabolic tissue damage and death [75]. Glucuronidation is important for detoxifying the bladder from toxins [76] and may be regulated differentially by fatty acid accumulation. While tissues, such as skeletal muscle and liver, are known to be highly susceptible to lipotoxicity [77], little is known regarding the effects of lipid accumulation in the bladder. Presumably, the functional impairment will occur in most healthy nonadipose tissues; however, this observation may not entirely apply to bladder tissue.

It may be possible that, in bladder tissue, lipid accumulation modifies metabolic functions in a way that strongly upregulates PPAR*γ* and enhances lipid uptake, similar to adipose tissue. Eventually, sufficient amounts of free fatty acids (FFAs) will be present in the bladder due to ectopic fat accumulation, and the bladder may no longer require A-FABP to import additional extracellular FFAs but will heavily utilize FAS for lipid production. FFAs bind PPAR*γ* and other PPAR isoforms and activate transcriptional activity. Other dysregulated metabolic pathways, including those that involve glycolysis [78], can cause a metabolic switch regulated by oncogenes and tumor suppressor genes to favor tumor growth and play a role in bladder carcinogenesis. Together, these observations are consistent with evidence showing lower expression levels of A-FABP and higher expression levels of FAS in more invasive forms of bladder cancer. Increased levels of PPAR*γ* activity may alter the microenvironment in a way that allows for the cells to autonomously synthesize nutrients within the bladder through lipid accumulation and angiogenesis. However, more studies need to be performed to understand the role of PPAR*γ* in bladder cancer.

## 6. The Impact of Dual-Acting PPAR Agonists

Despite evidence showing PPAR*γ* as the predominant PPAR in urinary bladder epithelium, PPAR*α* has also been found to be expressed in both rabbit and human bladder epithelium. PPAR*α* is activated by a class of synthetic ligands known as fibrates (i.e., fenofibrate) and is predominantly expressed in the liver, heart, brain, skeletal muscle, and kidney. Also, endogenous ligands such as fatty acids can bind PPAR*α* to increase transcriptional activity. Recently, bilirubin was also shown to function as an endogenous PPAR*α* agonist by direct binding [25] and was shown to decrease mRNA expression of PPAR*γ*. Once activated, PPAR*α* regulates genes that encode for mitochondrial and peroxisomal *β*-oxidation, which reduces dyslipidemia. In addition, activated PPAR*α* functions to hinder hepatic fatty acid synthesis through inhibition of FAS and SREBP1 and therefore lower lipid levels [21, 79, 80]. Dual agonists are a class of drugs that activate both PPAR*α* and PPAR*γ*, thereby combating diabetes mellitus and the metabolic syndrome among patients diagnosed with both conditions [81]. Examples of such dual agonists include ragaglitazar and muraglitazar, which would be of interests for the treatment of obesity and diabetes. However, muraglitazar has been shown to induce gallbladder adenomas in male mice, and ragaglitazar has been demonstrated to induce urinary bladder and renal pelvis tumors in both male and female rats [82].

It is worth noting that certain combinations of PPAR*α* and PPAR*γ* synthetic dual-acting agonists may have a carcinogenic impact on rodents, especially targeting urinary bladder epithelium. In a recent study, Egerod et al. found that rat bladder epithelium expresses both PPAR*γ* and PPAR*α* through a crosstalk link that involves the early growth response-1 (Egr-1) factor [18]. Egr-1 is a transcription factor and has been previously shown to play a role in bladder cancer among different species, including humans [83]. When either PPAR agonist is used alone, there is only slightly increased Egr-1 expression in the rat bladder epithelium [18]. High Egr-1 induction is dependent on the coactivation of PPAR*α* and PPAR*γ* by their respective synthetic ligands fenofibrate and rosiglitazone. Together, fenofibrate and rosiglitazone appear to exert a positive interaction in the bladder epithelium, upregulating high Egr-1 expression. However, this positive interaction is not observed in other tissues, such as the liver, where there are high expression levels of Egr-1 and the absence of carcinogenic effects of dual-acting agonists on rats [18].

It has also been demonstrated that ragaglitazar treatment has a carcinogenic impact on rat bladder epithelium and involves the induction of Egr-1 [82, 84]. Importantly, the fenofibrates that are PPAR*α* agonists have not been shown to induce bladder cancer. PPAR*α* agonists with a different structure, the clofibrates [85], have been shown to weakly enhance BBN-induced bladder carcinogenesis [86]. However, a second report indicated that clofibrates are not carcinogenic [87]. The differences in these studies may be from clofibrate potentially having off-target effects or through possible weak interactions with PPAR*γ*. Furthermore, it is rather a unique characteristic of bladder epithelium to express high levels of both PPAR*α* and PPAR*γ*. While the exact mechanism behind the interactions of PPAR agonists and bladder cancer remains unknown, these studies provide further insight into the relevance of PPAR activation, particularly in bladder cancer development.

## 7. PP5, a Positive Modulator of PPAR***γ***

**Figure 2:**
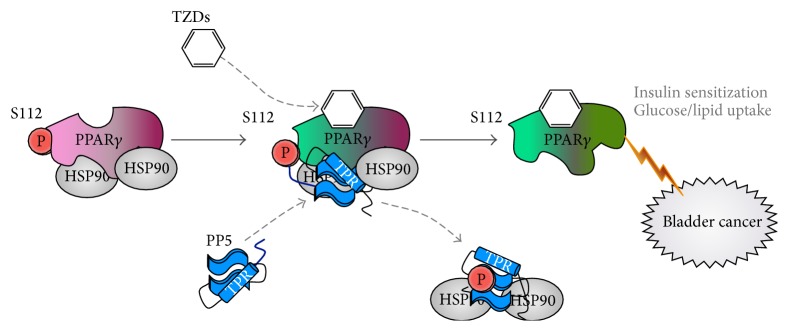
Theoretical model of PPAR*γ* and PP5 in bladder cancer. Activation of PPAR*γ* by TZDs recruits PP5 to positively modulate and dephosphorylate Ser-112 (S112). PPAR*γ* is activated once the phosphate group is removed, and a series of PPAR*γ*-mediated activities commence shortly thereafter, including insulin sensitization. PP5 has been shown to mediate PPAR*γ* activity by controlling phosphorylation of S112 in an adipogenic model, and targeting PP5 in the bladder epithelium may potentially affect PPAR*γ* and its carcinogenic effects on the bladder.

PPAR*γ* activity is inhibited by the phosphorylation of serine 112, and, currently, only one phosphatase, protein phosphatase 5 (PP5), has been shown to bind directly to the receptor [26]. PP5 belongs to the PPP-family consisting of serine/threonine protein phosphatases [88, 89]. Evidence has indicated that PP5 activation requires the binding of its tetratricopeptide repeat (TPR) domain to the heat shock protein 90 (Hsp90) chaperone complex [26, 89] (Figure 2). PP5 is a positive modulator of PPAR*γ* in the presence of proadipogenic activity, with PP5 described as a “prolipogenic phosphatase” [26]. Upon activation by the adipogenic stimulus rosiglitazone, PP5 is recruited to positively modulate the activity of PPAR*γ* by dephosphorylating PPAR*γ* at serine-112 residue [26, 90]. Once dephosphorylated, PPAR*γ* becomes active and regulates genes in metabolic processes, such as adipogenesis. Not only is PP5 a potential target in the treatment of obesity [26], but it may also provide an effective therapeutic intervention for bladder cancer. Other studies have suggested that PP5 plays a role in tumorigenesis. PP5 mRNA levels are remarkably elevated in malignant ascites hepatomas in rats [91]. Also, increased levels of PP5 protein have been observed in human tumor breast tissue and have been linked to the promotion of breast cancer development [92]. It is unknown whether a similar association exists between PP5 and human bladder cancer. The mechanism of PP5 expression and tumorigenesis has yet to be determined, but it may potentially regulate PPAR*γ* in the bladder epithelium similar to adipose, as high levels of PPAR*γ* are also associated with bladder cancer. If PP5 is a positive modulator of PPAR*γ* in the bladder epithelium, then reducing PP5 expression may serve as an alternative therapeutic target to hinder bladder cancer progression. However, these studies are yet to be conducted.

## 8. Conclusion

Long-term TZD therapy may increase the risk of developing bladder cancer, especially pioglitazone. Rosiglitazone does not appear to have the long-term effects on the bladder. Prolonged and higher PPAR*γ* activity levels are associated with higher incidences of bladder cancer, potentially due to the downstream effects of PPAR*γ*-mediated metabolism. In addition to incidence rates, PPAR*γ* activity is associated with increased bladder cancer cell migration and invasion. Further understanding of the roles of the PPARs and their agonists in the bladder may uncover additional strategies in bladder cancer therapy. Previously, there have not been studies examining the interaction of PP5 with PPAR*γ* in bladder epithelium and cancer development. It will be of therapeutic importance to determine if the same relationship exists between PP5 and PPAR*γ* in the bladder epithelium as for adipose tissue in the presence of TZD therapy. Bilirubin may offer a therapeutic potential because it activates PPAR*α* and suppresses PPAR*γ*, and fenofibrate has not been associated with bladder cancer. In the future, therapies that target PPAR*γ*, or possibly PP5, may prove to be useful in bladder cancer treatment, particularly among diabetic patients that require long-term health management.
